# Design, methods and populations of the CURV-24 study: a global systematic review and meta-analysis of 24-h urine volume and creatinine excretion in community-dwelling adults

**DOI:** 10.3389/fpubh.2026.1892695

**Published:** 2026-09-25

**Authors:** Espen Heen, Skjalg T. Kalvik, Sara Clarke, Rachel C. Davis, Saskia S. List, Kendal G. Chappell, Mekdes M. Kifle, Sigrun Henjum, Haakon E. Meyer, Ahmed A. Madar

**Affiliations:** 1 Department of Community Medicine and Global Health, Institute of Health and Society, University of Oslo, Oslo, Norway; 2 Department of Nursing, Faculty of Health and Welfare, Ostfold University of Applied Sciences, Fredrikstad, Norway; 3 Section for Study Skills, Department of Educational Support, University of Oslo Library, Oslo, Norway; 4 Department of Nursing and Health Promotion, Faculty of Health Science, Oslo Metropolitan University, Oslo, Norway; 5 Wings of Healing-Ethiopia, Addis-Ababa, Ethiopia; 6 Department of Physical Health and Ageing, Norwegian Institute of Public Health, Oslo, Norway

**Keywords:** 24-h creatinine excretion, 24-h urine volume, biomarker adjustments, community-dwelling adults, hydration status, meta-analysis, public health, systematic review

## Abstract

**Introduction:**

Urinary biomarker concentrations are increasingly used to assess intake, exposure or excretion, often by applying pre-specified 24-h urine volume values or normalization to creatinine based on prediction-derived estimates. However, both urine volume and creatinine excretion rates can vary substantially by sex, BMI, protein and salt intake, ethnicity, food culture, and short- and long-term climate. Ignoring such population-level differences may bias temporal, spatial, and cross population comparisons and undermine the validity of fixed reference ranges. CURV-24 is a global systematic review and meta-analysis designed to investigate this variability and its determinants in community-dwelling populations. The aim of the current article is to present the design and methods of CURV-24. We further describe the included studies and populations. Taken together, readers should be positioned to assess the quality and representativeness of the study data in the forthcoming meta-analyses.

**Methods:**

Using a broad, piloted syntax, we searched multiple bibliographic databases and registries (Medline, Embase, Web of Science, WHO Global Index Medicus, EBSCO Global Health, Cochrane, PROSPERO) without lan guage restrictions. Records published between 2015 and 2024 were investigated. We included primary studies with at least one timed 24-h urine collection from non-manipulated everyday conditions. Extensive efforts were made to identify companion reports and complementary datasets from authors and repositories. A tailored risk-of-bias and certainty-of-evidence framework, adapted from Grading of Recommendations Assessment, Development and Evaluation (GRADE), was developed to address population, collection, and analytical biases. The Preferred Reporting Items for Systematic Review and Meta-Analysis Protocols (PRISMA-P) and Meta-analysis Of Observational Studies in Epidemiology (MOOSE) have guided the development and reporting plans of the project.

**Results:**

The final dataset comprises 212 studies from 71 countries and 234 national populations, including approximately 270,000 individuals, of whom three-quarters were studied after 2010. About half of the studies examined more than 200 participants and one third used census-based probability sampling. Complete subpopulation data by sex, age, location, and ethnicity are available from a substantial subset of studies.

**Discussion:**

CURV-24 provides the most extensive synthesis to date of 24-h urine volume and creatinine excretion in populations worldwide. The project is positioned to improve hydration adjustment of urinary biomarkers, challenge fixed “one-size fits-all” assumptions and inform future best-practice guidelines in epidemiologi cal biomarker research. Looking ahead, CURV-24 has the potential to generate hypotheses on the relationship between climate and human hydration.

**Systematic review registration:**

https://www.crd.york.ac.uk/PROSPERO/view/CRD42024495900.

## 1 Background/rationale

Urinary biomarker research is a rapidly evolving field (1). The simplicity and non-invasiveness of a urine sample from a single void make it a preferred biofluid whenever reliable and valid measurements are possible (2). As a fluid of regulatory excretion, analytes that are kept within narrow boundaries in blood plasma may have earlier and larger signals in urine (3). The biomarker concentration can be compared to reference ranges or cut-offs (4). In epidemiological research, urinary biomarkers are regularly used in fields such as nutrition, environmental hazards, kidney disease, and hydration status assessment (3, 5, 6).

In 2022, our research group conceptualized what has become the study “24-hour urine volume and 24-hour urine creatinine excretion in general adult populations, a systematic review and meta-analysis”, hereafter referred to as CURV-24. The primary objective is to investigate the specific uncertainty introduced to urinary biomarker estimates of exposure, intake, or metabolism when interpretation is based on concentration alone as a proxy for an amount per time. In doing so, the central issue of urine dilution is not addressed (7). There is empirical evidence that the average urine volume over 24 hours (24 h Uvol) can be significantly impacted by sex, BMI, ethnicity, cultural habits, climate, geography, and study year in general populations (8–13). Yet, the global variability has, to the best of our knowledge, never been assessed or estimated systematically. Failing to take such population differences into account may invalidate temporal, spatial, and cross-population comparisons of biomarker concentrations and make the application of fixed concentration reference ranges questionable. Examples of urinary analytes for which such bias may be introduced into clinical and epidemiological assessments are iodine, fluoride, zinc, selenium, mercury, arsenic, phthalates, and bisphenol A, among others (14–18).

Secondly, CURV-24 seeks to investigate possible population-level predictors of average creatinine excretion per 24 h (24 h UCrEx), over and above the adjustments for sex, age, weight, and height, which are found in various equations that estimate 24-h creatinine excretion or convert spot urinary biomarker concentrations to daily excretion (19, 20). The motivation is the acknowledgment that creatinine-based prediction models that work well in one population may perform poorly in another (21, 22).

For each outcome, CURV-24 will explore possible associations with background characteristics measured at the population level: pregnancy and breastfeeding status, salt and protein intake, distinct cuisine, ethnicity, sampling year, season, geographical location, human development index, water quality and availability, and certain aspects of short- and long-term physical climate. Several of these covariates are considered proxies for social, physical, or physiological determinants affecting 24 h Uvol and UCrEx in humans. A more in-depth description of the rationale and the scientific underpinning for CURV-24 is outlined elsewhere (23).

Guidelines on how to perform a systematic review (SR) of observational studies exist (24–26). However, the development of search and inclusion strategies, as well as risk of bias (RoB) and overall quality-appraisal methods tailored to single-group continuous outcomes, are not well developed (26, 27). Furthermore, there seem to be few SRs presenting physiological estimators in the scientific literature, possibly because the classical strategy of defining the SR outcome as one of the inclusion criteria seems to be the norm. This, in turn, limits the potential number of included studies and the generalizability (28–32). To achieve its purpose, CURV-24 has therefore applied several methodological adaptations, which are briefly described throughout this manuscript. Further analysis and discussion of these considerations will be reported in a separate publication.

The aim of the current article is to present the design and methods of CURV-24 and to describe the included studies and the study populations from which all data originate, as a basis for judging the representativeness and quality of the empirical findings in the meta-analysis to come.

## 2 Materials and methods

### 2.1 Design

CURV-24 adheres to the PRISMA-P (33) and the MOOSE guidelines (34) and has been registered and made publicly available through PROSPERO (35) prior to data collection (36). A more comprehensive, time-stamped documentation of the study protocol and study progress with revisions is available at the Open Science Framework repository (23).

#### 2.1.1 Identifying the sampling universe and defining the inclusion criteria

The team identified research fields where the collection and measurement of 24 h Uvol are common (Table 1). To cover populations and world regions traditionally underrepresented in epidemiological biomarker studies, we included regional databases covering low- and middle-income countries (LMIC) with no restriction on article language (37–39). According to the World Bank Atlas method (as of 2026), LMIC are countries with gross national income per capita below $13,935 (40). In line with CURV-24’s overall aim, the team developed study inclusion criteria with the collection process of 24 h urine at the centre. This step is necessary to obtain the primary outcome of 24 h Uvol, which in turn is needed for calculating any 24 h UCrEx. In a condensed version, the primary study inclusion criteria were:

**Table 1:** Research areas where biomarkers are commonly measured in urine samples from 24-h collections.

Field of research	Examples of biomarkers and measurements
Macronutrients	Flavonoids, phytoestrogens, ketones, carnitine, nitrogen, ammonium, urea, amino acids
Micronutrients and minerals	Sodium (Na), potassium (K), chloride (Cl), sulphate (SO₄), phosphate (PO₄), fluoride (F), iodine (I), selenium (Se), B-vitamin metabolites
Human hydration measures	Urinary specific gravity, osmolality, urine colour, total water intake and loss, free water reserve
Heavy metal exposure	Lead (Pb), arsenic (As), mercury (Hg), cadmium (Cd), uranium (U)
Environmental hazard exposure	Phthalate, bisphenols, organophosphates, volatile organic compounds
Kidney health, hypertension, diabetes, (pre-)eclampsia	Urinary protein, albumin, creatinine, uric acid
Urogenital health	Voiding volume, voiding frequency
Hormones and neurotransmitters	Cortisol, sex hormones, adrenaline, noradrenaline, dopamine

“24h urine collection and Uvol measurement conducted in at least 30 adults aged ≥16 years, sampled from a general healthy, community-dwelling population under habitual daily living and without manipulation of physiological conditions.”

All primary study designs with at least one urine collection designed or timed to 24 h were includable. The process of 24 h urine collection could be related to an outcome, background, control, or intermediate variable. Study participants should not differ markedly from the surrounding general population: If the sample frame was a pre-selected group based on characteristics, disease, or overt risk factor for disease, the condition should be prevalent (>10%) in the general population and not expected to affect or be associated with physical functioning, nutritional aspects, water turnover, or urine production. The hydration physiology should represent a steady state. Equally important was to ensure that the relevant data represented individuals in their everyday life, with as little interference (non-manipulation) as possible. Pregnant and breastfeeding women were included as an exception to the physiological rules above and constituted two unique subgroup. Specific medical conditions, characteristics and contexts deemed compatible or incompatible with inclusion are described in Supplementary material I.

If available, the mean of repeated individual measurements across days or weeks was preferred as a better proxy for long-term exposure. In open cohorts with collection points at least 3 years apart and with at least 50% new participants, each time point was treated as a separate primary population while retaining its common cohort origin.

The outcomes of 24 h Uvol or 24 h UCrEx should either be explicitly reported in at least one publication from the underlying primary study or be possible to obtain from the research group responsible for the study data. As such, we were primarily looking for supportive information that 24 h Uvol was collected, even if not reported. Since our interest was in implementing our findings to the current public health and research needs, the team restricted the earliest primary data collection to be included in analyses to 1990.

#### 2.1.2 Considerations on variability, dependency, error and bias

The variation in population means of 24 h Uvol and UCrEx is best conveyed through prediction intervals, which are expressed on the same scale as the raw data (41). Such an unadjusted range across healthy adult populations globally reflects the variation in mean urine volumes and creatinine excretion that is implicitly incorporated into the calculations and interpretations of biomarker excretion in many primary studies. A related, yet more challenging, question concerns the biological variation in these population means. Addressing this requires closer examination of the inaccuracy (bias) and imprecision (error) that inherently affect such study outcomes. The contextual variables and the analysis plan have been designed to explore both perspectives.

CURV-24 is primarily concerned with variation between populations rather than between studies. In this respect, a population is a defined observational unit of individuals that can be characterized in a meaningful way for the purpose of epidemiology. In our planned meta-analyses, the primary analytical unit will therefore generally be the most disaggregated subgroup for which the outcome and relevant background characteristics are available and refer to the same participants. Estimates originating from the same study or cohort will remain linked in the analytical structure, and their dependency will be accounted for using multilevel random-effects models. For overlapping subgroup estimates, mutually exclusive subgroup sets will be preferred where possible; otherwise, their correlation will be accounted for. This also applies to repeated observations from the same cohort.

The interpretation of pooled estimates will follow the same population-oriented perspective. An unadjusted random-effects meta-mean will describe the average across the types of populations represented in CURV-24 and will not be interpreted as the population-weighted mean 24 h Uvol or UCrEx of all adults globally. Geographical location and other population characteristics will instead be explored as possible sources of between-population variation through stratified analyses and meta-regressions. If an explicitly population-weighted global estimate is presented, the target population and weighting strategy will be defined separately.

#### 2.1.3 Needed number of population estimates and availability of relevant data

For CURV-24 to provide findings that will be relevant for the research community, a precision in the meta-analytical metrics within 10% of the point estimate is desirable. This translates to a 24 h Uvol difference of 0.1–0.2 L, a magnitude of error and bias unlikely to make any real difference for epidemiological interpretation of biomarker levels in urine. We conducted a simulation to examine how the number and sample size of included populations would affect the precision of the primary outcome of 24 h Uvol. The population mean and SD of 24 h Uvol were extracted from the INTERSALT study of 52 geographically distinct populations (42). Although this multi-country study was performed in the late 1980s and may not represent the current global situation, we expected the variance around the means and meta-means in INTERSALT to be the best proxy for what we would find in CURV-24. Focussing on a random-effects meta-distribution, the desired precision in a 95% prediction interval of population means was estimated to require somewhere between 20 and 25 independent population estimates, each of *n* ≥ 30.

The team investigated recently published SRs in the relevant fields of research. The assessment indicated a probable number of included studies between 100 and 300 from all regions of the world, well within the minimum sample size and likely to provide enough power to compare subgroup prediction intervals.

#### 2.1.4 A modified framework for assessing the certainty of evidence

Our team has not found a standard GRADE approach appropriate for CURV-24’s design (43). We therefore developed a modified certainty assessment that integrates the five common dimensions of a GRADE process and defines assessment strategies (Table 2).

**Table 2:** Dimensions in the assessment of the certainty of the evidence produced in CURV-24.

Dimension	Assessment strategy	Concern	Certainty of the evidence
Indirectness (sampling frame and process)	Conceptual model vs. CoCoPop^a^ (broad/narrow exclusion criteria) Sensitivity analyses	No serious concerns (0) Serious concerns (−1) Very serious concerns (−2)	High Moderate Low Very low
Risk of bias (urine collection, creatinine analysis)	Sensitivity analyses External evidence Direction of bias		
Imprecision	The confidence interval of the meta-mean and the prediction interval (.) Uncertainty vs. minimal relevant difference (0.1−0.2 L) or relevant clinical cut-off Risk of inflation of the prediction interval		
Inconsistency	Unexpected variability compared with conceptual model. NB: ecological and atomistic fallacy Shape of the meta-distribution of the outcome Meta-regressions at subgroup level (size and direction of effect modifiers)		
Publication/small study bias	Funnel plot by sample size and SD Ratio of eligible studies lacking outcome reporting to all included studies and comparison of study characteristics		

^a^ Condition, Context and Population (JBI).

In this assessment matrix, study risk of bias (RoB) starts at a high certainty rating, as all types of studies have the potential for high-quality descriptive data. For each dimension assessed, serious or very serious concerns can downgrade the final certainty in the evidence (high to very low) one or two levels. The GRADE principles are drawn from a recent in-depth article series (44).

### 2.2 Methods

#### 2.2.1 Search strategy, database syntax and quality assessment of eligible reports

Many articles report 24 h Uvol or UCrEx values without explicit mention in the title, abstract, or keywords. This prompted us to operationalize the search syntax into four mandatory *inclusion concepts:*

(24-h) AND (urine or void or volume or output or sample or collect or excret* or creatinine or “the specific urinary biomarkers by name”) AND (general population or pregnant or breastfeeding) AND (adults aged ≥ 16 years).

The search syntax was developed on the Ovid platform and tested in Medline. Each concept was broadened to cover synonymous terms and terminology that could indicate a collection of 24 h urine by methodological convention, such as “sodium intake” or “urinary cortisol excretion”. This was achieved by MeSH (Medical Subject Headings) exploding and investigating relevant records for conceptual terms. Filters removing “children,” “animal studies” and “non-relevant article types” were modified from existing filters developed by the literature search team at the UiO Library and applied.

A pilot corpus of articles was established from the 100 “most relevant” records of the first explorative Medline search, enriched with pre-collected articles reporting population data on 24 h Uvol or UCrEx from several research fields and indexed in PubMed between the years 1990 and 2023. In total, 140 reports were assessed and 44 met all principal inclusion criteria. The pilot corpus was used to test the sensitivity of the search syntax. For new versions, the team analysed what caused a particular relevant record not to be picked up by our title and abstract search and modified the syntax accordingly. The specificity of the syntax was improved through assessment of clearly irrelevant records retrieved during exploratory searches. For each inclusion concept, a set of keywords that negated such a concept, like “spot urine,” “intensive care patients”, “dialysis”, “incontinence” or “paediatric” were listed (Supplementary material II). The Endnote search functions identified the approximate number of titles and abstracts that scored high on a combination of such words and therefore would have a low probability of being included in future systematic screening. The sensitivity increased from approximately 70 to 90% in the final version. We judged this level to be the best trade-off between loss of relevant studies and screening workload of obvious irrelevant studies.

Guided by studies that have assessed citation indexing overlap across health-related scientific databases, records in Medline, Embase, Web of Science, WHO Global Index Medicus (all regional databases), EBSCO Global Health, Cochrane database of systematic reviews, and PROSPERO register were included in the final search (37–39). The syntax was translated to all databases and peer-reviewed by an independent research librarian, using the PRESS 2015 guidelines (45). The final syntax with annotations for all eligible databases can be found in Supplementary material III.

#### 2.2.2 Software for managing the SR

The team investigated and tested the capabilities of Cadima, Rayyan, Eppi-reviewer, JBISUMARI, and Covidence. The latter became the study platform. The web application had a suitable combination of a user-friendly interface, possibilities for tailor-made data-extraction forms, effective administrator tools, a broad tutorial library, and fast handling of questions through the customer-service team. Safe data backup and work logs were equally important. Translation tools for non-English-language reports were investigated. The software DeepL Translator (2025) was able to work directly from pdf to pdf and became the principal software per protocol, whereas Google Translate (2025) was the best tool for HTML documents. In some languages, Google Translate performed better. Reports in Chinese and Persian were poorly translated by AI tools. We involved native-speaking research colleagues to assist in translation of these two languages.

#### 2.2.3 Piloting, standardization and equipping the team

The screening and data-collection team consisted of the principal investigator (EH) and four co-researchers (RD, SL, KC, MM), each with formal SR training. A pilot project was created in Covidence. Combining the pilot corpus with additional eligible reports, 472 records were screened by title and abstract and 328 were excluded, 21 full texts were assessed for eligibility, and 15 studies were included and scrutinized for relevant data. The piloting supported development and testing of the data extraction form and the *Guide for screening and data extractions,* containing procedures, definitions, and recording rules for each item/variable and details of how to handle nonconforming reporting. The guide was revised twice during the initial screening work. All versions can be found in the OSF repository (23).

Excluded studies were categorized (Table 3). Systematic, scoping, and narrative reviews were not included, but they were hand-searched for additional references before exclusion per protocol.

**Table 3:** Definitions of study exclusion reasons in CURV-24.

Exclusion reason		Explanation
1	Review^a^	Refers to any type of review.
2	Population <16 years	Populations with individuals under 16 years.
3	Non-free-living population	Populations from hospitals, prisons, research facilities, and other confined settings.
4	Diseased population	That may distort representation of the general population.
5	Condition affecting outcome	Such as manipulated diet or intravenous fluid administration. Does not include pregnancy/breastfeeding.
6	*n* < 30	The includable population as described in reasons 1 through 5 must have a minimum of 30 individuals.
7	Outcome data not collected	Studies which did not measure or collect all urine produced in 24 h.
8	Outcome data not reported	24 h urine volume measured but unreported.
9	Awaiting classification	Insufficient information to determine eligibility.

^a^ Each was screened for relevant records.

The pilot screening gave a title and abstract inclusion rate of approximately 25%. Starting with an expected 20,000 eligible records, this translated to an unreasonably large workload for a team of five. We decided to provide flexibility by first screening the records published in the last 5 years and then continuing with older five-year strata in a stepwise manner until the needed study number was reached. In this way, there would be several potential exit points without losing global integrity and validity.

To increase the team’s work capacity, we explored generative AI as one of the reviewers in full-text screening in an auxiliary research project. Based on the kappa-score, we did not achieve a comparable screening quality to human reviewers, but the AI model increased screening speed when used as an assistant to the second reviewer and was used as such. This part of the methodological work has been published elsewhere (46).

#### 2.2.4 Data acquisition, extraction and quality appraisal

The pilot-screening indicated about 70% non-reporting of the CURV-24 outcomes among includable reports. Such studies would temporarily be labelled “awaiting classification,” remaining at the full text screening stage for further investigation of companion reports. A companion report originates from the same underlying study and could itself be an included report or found through reference lists and other bibliographic investigations. If still unresolved, authors of the included reports were contacted. Per protocol, we prioritized studies from countries or regions with no or few other included studies, with subgroup data, with more than 100 participants, and/or with probability sampling from the general population.

Additionally, data repositories were searched for relevant data, and corresponding author(s) and study administrators were requested to share population mean (SD) values for 24 h Uvol, 24 h UCrEx, age, weight, height, and BMI if not already published. If the study population could be subdivided by sex, age-group (<39, 40–65, 65+), ethnicity, or location, we asked for subgroup data. Some reports did not convey enough information to decide on inclusion/exclusion. In these instances, our request was primarily related to the determination of eligibility and secondarily to variable reporting. At a later stage, the authors of the included studies were re-contacted to obtain additional contextual information for the quality appraisal. All inclusion/exclusion decisions were made by two independent reviewers and discrepancies resolved by consensus.

The data extracted are presented in a condensed form in Table 4. Continuous outcomes and covariates were extracted in raw form, including the number of observations (n), mean, measures of dispersion (SD, SEM, or CI), or percentiles (P0, P25, P50, P75, P100). Whenever possible, unadjusted values were preferred over adjusted or normalized values (e.g., per kg body weight, per g of creatinine). A representation of the data extraction form can be found in Supplementary material IV.

**Table 4:** Key study variables captured in CURV-24.

Study and population variables
Study-level themes
Bibliographical data, Study ID, Subpopulation ID, Main field of research, Corresponding author and email, Aim of the study, Study primary outcomes, Study design, Country/region/place, Start/end month and year, Number of relevant subgroups
Outcomes
Primary: 24-h urine volume. Secondary: 24-h urinary creatinine excretion.
Continuous covariates
Age, Height, Weight, BMI, Urinary osmolality, Urinary specific gravity, Urine colour scale, Urinary Na excretion, Urinary K excretion, Urinary iodine excretion
(Sub)population predictors
Place, Ethnicity (10 cat.), Sex, Fertility status for females (pregnant/breastfeeding/none/unknown), Socio-economic status within the community (high, medium, low).

The team collected data about each study’s internal and external risk of bias across three dimensions: (1) deviation from the ideal sampling frame (sample selection bias), (2) urine collection bias, and (3) creatinine analytical bias. The assessment was built on the JBI (formerly the Joanna Briggs Institute) nine-item tool for quality appraisal in observational studies with considerable modifications (47, 48). The items in the assessments are found in Table 5, with further details in Supplementary material V.

**Table 5:** Variables collected for the purpose of quality appraisal.

Quality appraisal variables
Participant sampling
Recruitment strategy, Population sampling frame, Participant inclusion criteria, Participant exclusion criteria, Sampling method, Participant selection bias, Balanced drop-out.
24 h Uvol collection and measurement
Collection day, Organisation of collection, Appropriate collection method/measurement, Instruction in urine collection, Testing of participant comprehension, Participant support, Observed time of collection, Exclusion of study participants or urine, Adjustment of reported urine volume, Sensitivity analysis and reliability assessment of mean volume.
Creatinine analysis
Sound collection/laboratory manual followed, Method of creatinine analysis, Instrument name/type, Laboratory bias and error measures (63), Sensitivity analysis and reliability assessment of creatinine values.

#### 2.2.5 Statistics

##### 2.2.5.1 Sample size calculation

The complete INTERSALT study of 52 populations, each with 200 individuals, had a male 24 h Uvol meta-mean confidence interval of ±80 mL. The team investigated the 95 percent prediction interval (PI)—the range within which a single population mean will fall 19 of 20 times when drawn randomly from a universe of similar populations. Keeping all other 24 h Uvol parameters constant, a smaller sample of 20 populations, each with 30 participants, reduced the precision of the meta-mean by an additional ±4 mL and widened the PI by approximately 200 mL, approaching the minimum clinically relevant difference per protocol. Reducing the average number of participants below 30 quickly reduced the absolute precision. The number of independent populations was more important for the precision than the average number of subjects per study. Individual population estimates based on >200 participants had a precision in the mean 24 h Uvol of approximately ±100 mL and could be meaningfully compared statistically. The simulation was performed using the *Comprehensive Meta-analysis* software (49).

##### 2.2.5.2 Standardisation, transformation, and pooling of continuous data

Continuous variables were often reported in percentiles and had to be converted to mean and SD. Different methods for estimating the mean and SD have been proposed (50, 51). Authoritative SR guidelines do not seem to include recent statistical developments (52). The team therefore established a standard procedure for the situation of estimated normal, transformed-normal, and unspecified populational distributions. If summary statistics were unlikely to have come from a normal distribution (*p* < 0.05), we used estimations based on quantile estimation, Box-Cox transformation, or the” unknown non-normal distribution” models, developed by McGrath and colleagues (53, 54). When normality was justified, the Luo/Wan method presented by Tong and colleagues was applied (50, 55–57). Web-based applications were available for the four methods (58, 59). The two main outcomes showed a mix of normal and non-normal population distributions, indicating little skewness on average. The model performance across 26 cases, several reporting both mean (SD) and median (percentiles), showed mean estimates close to the empirical mean regardless of the model used. The SD values were 13–18% larger on average with the models for skewed versus normally distributed data. The transformation decision tree is available in the OSF repository (23).

To avoid double-counting of subjects in meta-analyses, mutually exclusive subgroup data were pooled using standard equations with subject n, mean and SD (60). Reported concentration and excretion values, or concentration and Uvol values, were not combined to derive CURV-24 outcomes due to lack of data necessary for supporting statistical assumptions or covariances.

##### 2.2.5.3 Agreement between the reviewers

The duplicate screening of titles and abstracts had a proportionate agreement ranging from 0.90 to 0.96 and Cohen’s Kappa between 0.67 and 0.87. For the three reviewer-pairs with more than fifty comparisons in the full-text review, the proportionate agreement ranged from 0.993 to 0.996, and the Kappa score between 0.89 and 0.98.

##### 2.2.5.4 Descriptives of the included studies

Categories are presented as frequencies, ratios, or percentages, rounded to a level of appropriate accuracy. Formal categorical count testing was done with Fisher’s exact test because some cell counts were below five. Skewed continuous data are presented as median (IQR) and min-max. Trend analysis of primary report publication years was restricted to 2020–2024 and performed using standard Poisson regression, with trend reported as the antilog (e) of the beta-coefficients. All descriptive analyses and formal tests include missingness (other, unclear, system-missing). If the year of urine collection could not be retrieved, we assumed a date 36 months before the publication date of the earliest retrievable companion report from the study. A two-sided probability of <0.05 was considered significant. The analyses were performed in STATA/SE 19.5.

## 3 Results

### 3.1 Search and screening

The final search strategy (run 02 Jan 2024) retrieved all records published after 1990, yielding 21,891 unique citations after automated and manual de-duplication. An additional search on 12 Sep 2024 added 785 new records. From this pool, and in accordance with our sample size requirements and screening strategy, records published 1 Jan 2020 onwards resulted in a sufficient number of included studies. In the pregnant-and-lactating subgroups the second time stratum starting 1 Jan 2015 was needed to meet the minimum sample size requirements. After removing retracted papers, 5,217 records remained for screening. Reports in Chinese, Finnish, French, German, Japanese, Korean, Persian, Portuguese, Russian, and Spanish were translated into English. In fewer than 10 instances, the principal investigator determined inclusion/exclusion when the two original reviewers could not reach a consensus. The PRISMA diagram in Figure 1 shows the workflow.

**Figure 1:**
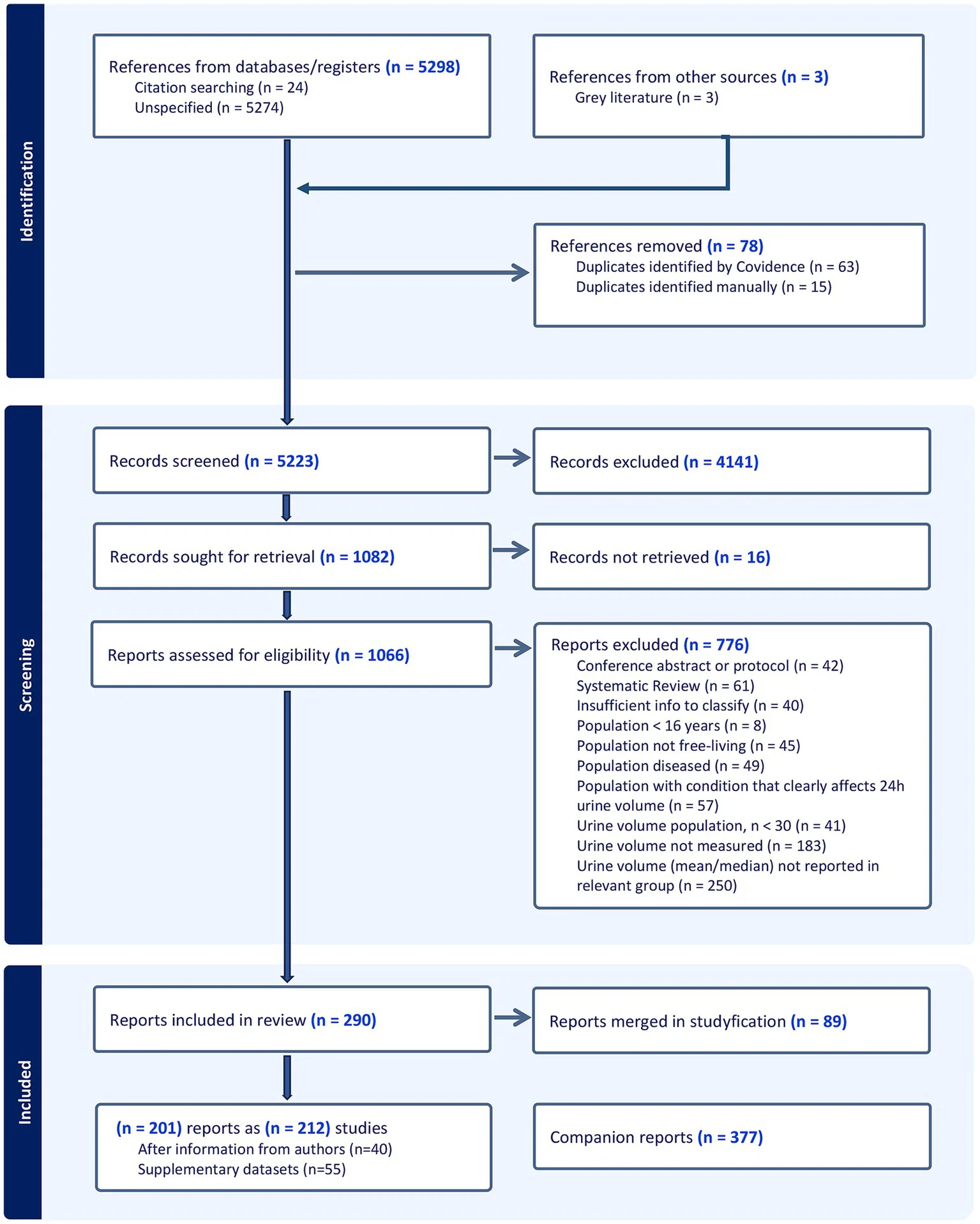
The PRISMA diagram showing the flow to final study inclusion. The exclusion reason “Insufficient info to classify” was related to the inclusion and exclusion criteria. The 377 companion reports were added during data extraction. Chan 2022 (INTERMAP) represents four separate national studies; Ma 2022, Magnusson 2020, and Tan 2022 three studies each; Choi 2020 and Eensoo 2023 two studies each.

We screened 36 SRs with potential for relevant reports published between 2020 and 2025 and 24 additional records were included. The 3 records from grey literature were shared by collaborating research groups. In total, 540 reports had the right population, context, and 24-h urine collection. For 250 of these, CURV-24 outcomes were not possible to retrieve, leaving 290 included reports. During the process of defining unique studies, 89 of these were merged before data-extraction. The number of added companion reports was 377.

Authors from 153 studies were contacted to obtain additional information or datasets. These communications resulted in eligibility determinations for 70 studies, of which 40 studies were ultimately included. Complementary numerical data were obtained from research groups and/or data repositories in 55 instances, or in 46 instances when restricted to studies containing subgroup data.

### 3.2 Study characteristics

The search strategy and identification methodology have resulted in 212 studies from 71 countries on all habitable continents with data from 234 unique populations and 1,043 subgroup (Table 6). The mean number of subgroups per study is five and the maximum within a single study is 26. Low- and middle-income countries constitute 44% of all studies. Eighty percent of the studies have included both sexes (Table 7). Within-study subgroups stratified by sex, age, location, and ethnicity are available in 111, 47, 23, and 13 studies, respectively. In 23% of studies, 24 h Uvol or 24 h UCrEx served as study outcomes or predictor variables, whereas the remaining studies treated them as intermediary variables.

**Table 6:** Number of countries and number of studies per country, organized by continent.

Asia/Oceania		Africa		North America		South America		Europe		Middle East	
Bangladesh	2	DR Congo	1	Belize	1	Brazil	4	Belgium ^e^	2	Iran	8
Cambodia	1	Ghana	1	Canada ^e^	4	Chile	1	Croatia	2	Israel	1
China ^e^	31	Morocco	2	Costa Rica ^e^	1	Paraguay	1	Czech Republic ^e^	3	Oman	1
India	5	Nigeria	2	Dominican Republic ^e^	1	Peru	2	Denmark	2	Palestine	1
Indonesia	2	Somaliland	1	El Salvador ^e^	1	Uruguay	1	Estonia	2	Saudi Arabia	1
Japan ^e^	9	South Africa	4	Guatemala ^e^	1			Finland	1	Türkiye	5
Malaysia	2	Tanzania	1	Honduras ^e^	1			France ^e^	1	UAE^d^	2
Mongolia	2	Tunisia	1	Mexico	3			Germany ^e^	8		
Nepal	2			Nicaragua ^e^	1			Greece ^e^	1		
Pakistan	1			Panama ^e^	1			Hungary ^e^	1		
South Korea	3			United States ^e^	26			Italy ^e^	5		
Sri Lanka	1							Kosovo	1		
Thailand	5							Lithuania	1		
								Netherlands ^e^	14		
								Norway ^e^	4		
Australia	3							Poland ^e^	2		
Fiji	1							Portugal	4		
New Zealand	2							Slovenia	1		
								Spain ^e^	2		
								Sweden	4		
								Switzerland	9		
								Romania ^e^	1		
								Russia ^e^	1		
								UK^c^	7		
16^a^	72^b^	8	13	11	41	5	9	24	80	7	19

^a^ Number of countries and ^b^ number of study populations per continent, ^c^ The UK is represented by populations from England, Scotland, Wales, and Northern Ireland, ^d^ United Arab Emirates, ^e^ Seven studies were multi-country and split into national populations.

**Table 7:** Basic characteristics of the included studies.

Study characteristics	Frequency	Percent
Income country		
High	119	56.1
Upper middle	71	33.5
Lower middle	20	9.4
Low	2	0.9
Main field of research		
Salt intake	79	37.3
Kidney-urogenital	29	13.7
Nutrients^a^	29	13.7
Hypertension, pre-eclampsia	27	12.7
Iodine nutrition	20	9.4
Hormones/neurotransmitters	10	4.7
Hydration	10	4.7
Environmental substances and hazards	8	3.8
Study design		
Cross sectional study	129	60.9
Longitudinal prospective/cohort study	42	19.8
Clinical trial	13	6.1
Diagnostic test validation	12	5.7
Case–control study	8	3.8
Non-randomized experimental	5	2.4
Other	3	1.4
Type of sampling frame		
General, healthy selection	87	41.0
General, not the complete population^b^	57	26.9
General, census^b^	53	25.0
Selected with common and mild disease	13	6.1
Unclear	2	0.9
Method of participant sampling		
Convenience	92	43.4
Probability	64	30.2
Unclear	30	14.2
Systematic	16	7.6
Quasi-probability sample^c^	10	4.7
Sex of study population		
Both	170	80.2
Female only	37	17.5
Pregnant, breastfeeding subgroups^d^	(25)	(11.8)
Pure pregnant, breastfeeding studies^e^	(21)	(10.0)
Male only	5	2.4
Total^f^	212	100.0

Related categories with few occurrences are collapsed. Missingness (unclear, other) is included as a category. ^a^ Other than NaCl and iodine. ^b^ Combined as “general population sampling frame”. ^c^ Failed attempts with supplemental recruitment or pooled population studies with different participant sampling. ^d^ With a total of 77 subgroup of pure pregnant or breastfeeding, of which ^e^ 21 studies consisted only of these subgroup. ^f^ The total number of studies and the sum of percentages within each category.

Ten reports published before 2020 are from the subgroups of pregnant and lactating women. There is a significant reduction in the included primary reports published between 2020 and 2024, on average 16.3% (CI 7.5–24.3%) per year (Poisson regression of counts, LR chi2(1) = 12.40, *p* < 0.001). In the included studies, urine was collected between 1985 and 2022 (median 2016) and predated publication of the primary report by a median (IQR) of 6 (4, 10) years. Urine collection commencing after 2010 constitutes 77% of all studies (Figure 2).

**Figure 2:**
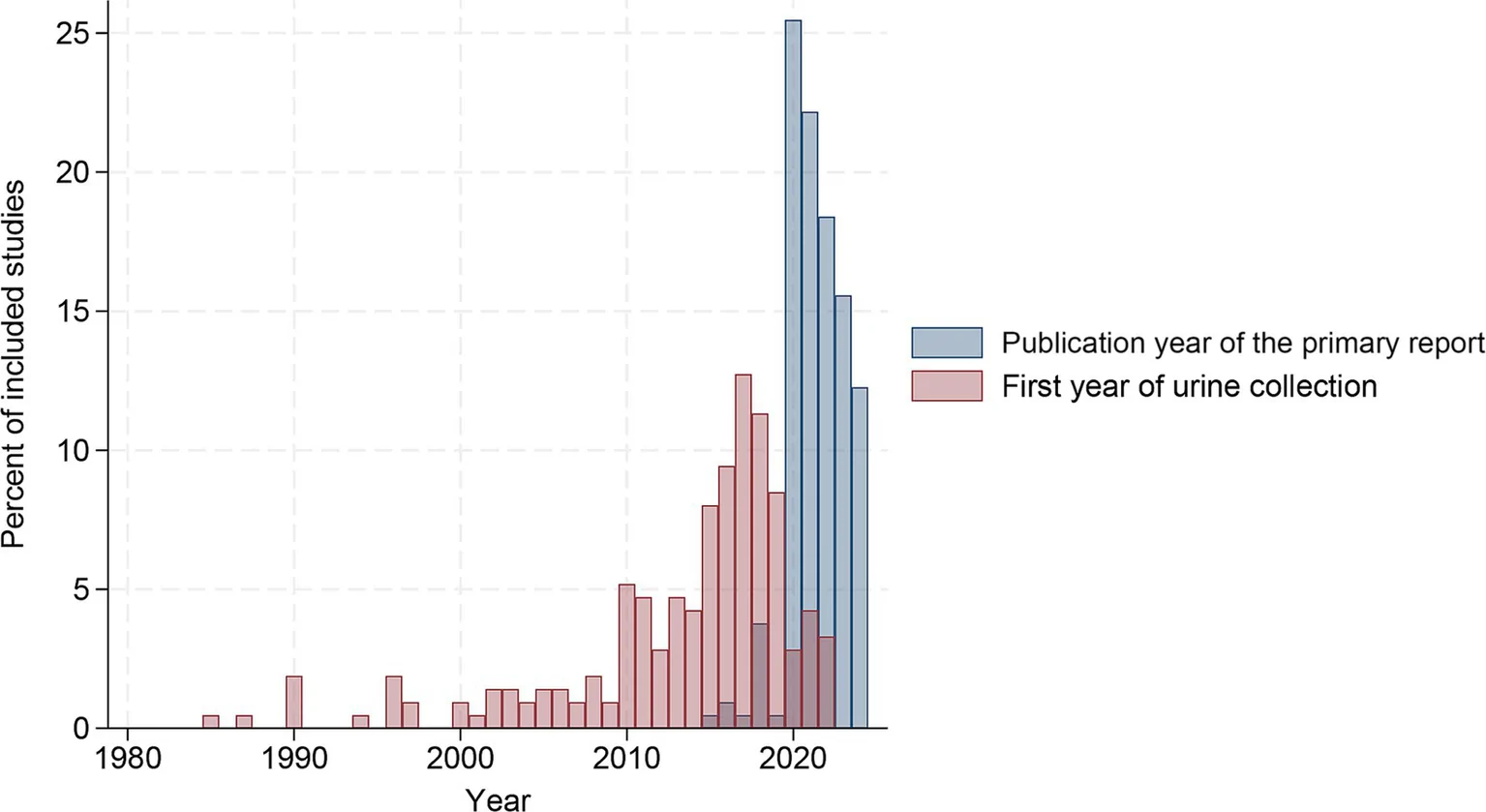
Distribution of publication year of primary reports and urine-collection year for the 212 included studies. Urine collection was set to 36 months before publication of the earliest report from the study in 26 instances. The publication years 2015–2019 constitute 6%.

Table 7 shows a predominance of studies related to salt intake/excretion (37%). All pre-identified research fields have contributed to the overall number of studies. Most studies are of cross-sectional design (61%), followed by longitudinal prospective/cohort studies (20%).

The population sample frame ranges from national census registries to small convenience samples. For “salt intake” studies, 64% (50/79) have a sample frame approximating an unselected general population. Similar numbers for “hypertension” and “kidney-urogenital” studies are 56% (15/27) and 41% (12/29), respectively.

Overall, 30% of studies have used some form of probability-based participant sampling, with salt studies showing the highest proportion at 56% (36/64). By contrast, 43% of studies have relied on convenience sampling of subjects. A significant clustering is observed between sampling frame and method of sampling: studies with a general population sampling frame more often utilized probability sampling (66/110), whereas studies restricted to healthy individuals or those with mild disease were more likely to use convenience sampling or had an unclear sampling method (85/100). In 14% of studies, the sampling method could not be determined (crosstabs and Fisher, not displayed).

### 3.3 Participants

The median (IQR) number of participants across studies is 256 (91, 744), ranging from a minimum of 30 (lower inclusion criteria) to almost 120,000 subjects. The total number with outcome data included in the systematic review is 272,906 individuals. After removing the largest study, the second largest has 13,701 participants, the median (IQR) sample size reduces to 250 (88, 743), and the total sample size becomes 153,412 participants (Figure 3). Of these, 94,870 individuals were studied after 2010 and 73,008 individuals are from studies that we consider to represent the general population with overall low risk of sampling and selection bias.

**Figure 3:**
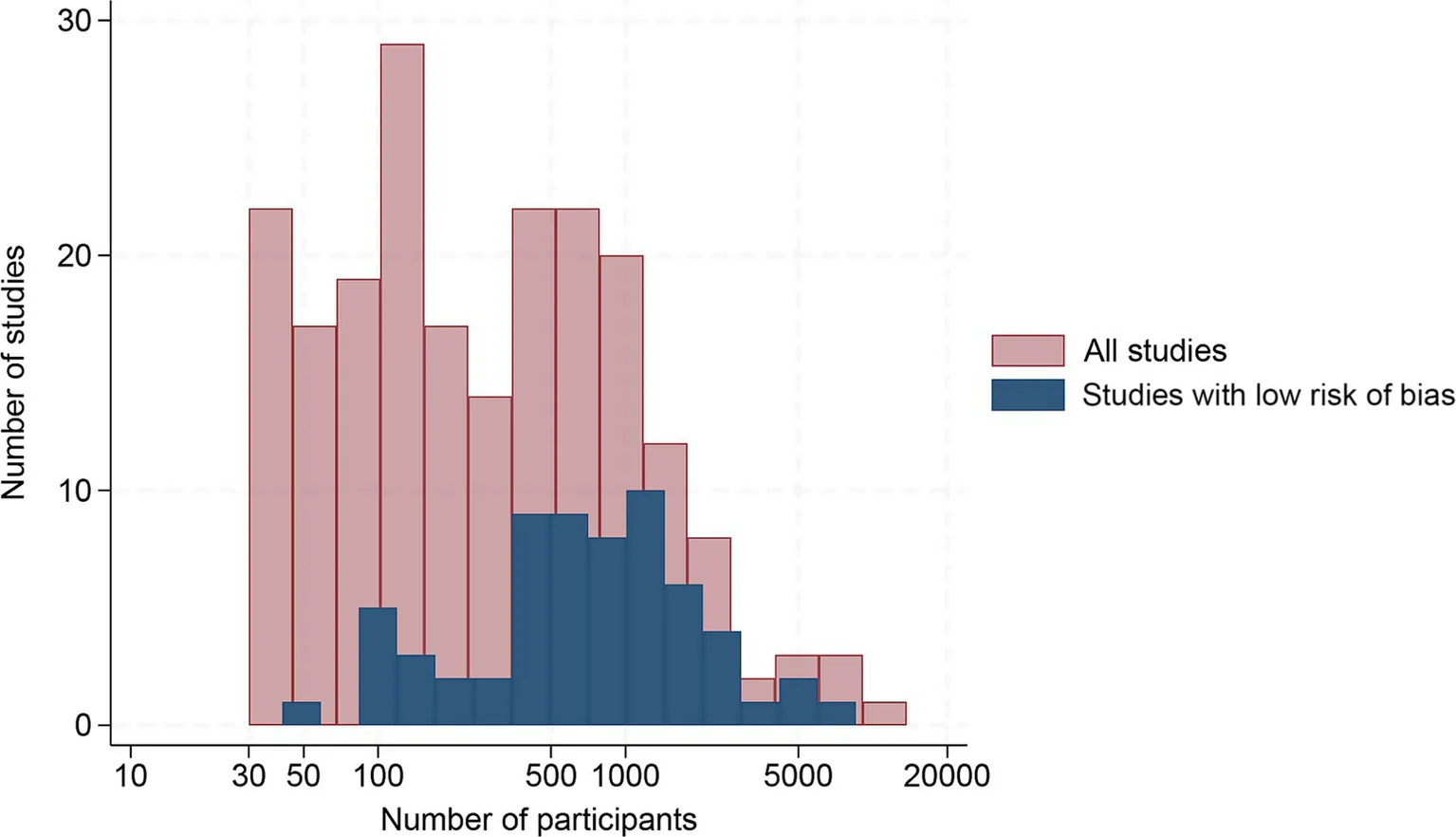
Number of studies by study sample size. The *x*-axis is compressed to the natural logarithm (*ln*) scale of the study sample size. The largest study (120000) is not displayed but is in the category of “low risk”.

## 4 Discussion

In this article, we have presented the purpose, design, and methods of the CURV-24 project. The characteristics of the 212 included studies show a substantial diversity in geographic origins, research fields, and study types. CURV-24 has surpassed the minimum number of studies required to answer the main goals of the project and will have sufficient power to conduct meaningful subgroup analyses according to sex, pregnancy and breastfeeding status, age, anthropometrics, location of study, and ethnic characteristics.

### 4.1 Design and methodology

A systematic review (SR) with the goal of estimating physiological parameters across populations is conceptually different from the classical SR aimed at establishing the effect of an intervention (27, 61). When natural variability between populations (random effects) is to be expected and explored, the estimate of dispersion (variance, prediction interval) is often more important than the pooled effect size (41). Therefore, we have attempted to cover a wide variety of contexts within the boundaries of inclusion.

The objectives in CURV-24 represent two layers of complexity. At the outer layer, the study aims to describe the heterogeneity in 24 h Uvol and UCrEx as they are measured and reported across populations. We may further adjust for population composition and population- and study-level covariates to make the outcomes directly comparable. At the inner layer, pure biological variation is at the center of investigation. We expect the inherent bias of sampling, collection, and laboratory measurements to obscure this true biological variation. The CURV-24 design has clear limitations in delineating these variabilities. Cross-level bias makes it difficult to differentiate between individual and population-level effects. Some of this can be addressed by including the existing within-study between-subgroup level in the analysis. We have specified the analytical principles, but the exact statistical model will depend on the structure, completeness, and dependency of the populations available for each analytical question. Additionally, routine measurements of creatinine concentration are prone to bias. The assessment of the biological variation in UCrEx will therefore require sensitivity analysis of high and low risk of bias related to the creatinine analytical method (62, 63).

Whereas many studies provide only one overall population estimate, others provide numerous estimates by sex, age, location, ethnicity, or other characteristics. Similarly, some countries contribute considerably more populations than others. If every available estimate is treated as an independent observation, studies and countries with more detailed reporting could receive disproportionate influence. Conversely, reducing every study to a single estimate would discard much of the population-level variation that CURV-24 was designed to investigate. Retaining subgroup information while accounting for its dependency provides a way of balancing these concerns. The exact implementation will therefore vary according to whether an analysis involves distinct subgroup, overlapping subgroup summaries, or repeated observations of the same cohort.

In particular, the geographical breadth of CURV-24 should not be interpreted as global representativeness. A pooled global or regional meta-mean will primarily summarise the populations represented in the available evidence. We will therefore place greater emphasis on the spread in population means, the prediction intervals, and the associations with the covariates rather than on average point estimates.

It could be argued that bringing primary study covariates into CURV-24 as outcomes may be less rigorous and more prone to bias compared with pure outcome extraction. We have tried to mitigate this risk by extensively collecting contextual information for quality appraisal. Whether sampling and selection bias materially affect the CURV-24 outcomes in the first place is unclear, given the physiological nature of our outcomes. Furthermore, the risk of publication and selective outcome bias, which is linked to a study’s outcome, may be reduced with the current design.

In CURV-24, an analysis of possible systematic deviations from the ideal population, context, urine collection method, and accuracy in measurements informed the risk-of-bias items that we extracted. Accordingly, less emphasis was placed on assessing the study design or following predefined items in related SR guidelines (47, 48). In general, SRs of studies measuring physiological parameters demonstrate uncertainty about how to formally assess risk of bias, even in SRs of high quality (64–69). Additionally, there is a paucity of formal assessment of the certainty of the evidence in these SRs. This is not surprising, given that most established evaluation frameworks were developed primarily for randomized controlled intervention studies. Our team has therefore chosen to develop our own evaluation framework, drawing from the principles of the five GRADE dimensions (44). We believe a meta-evaluation is important to any SR, regardless of the type of outcome. By describing the principles behind our assessment of the certainty of evidence up front, the subjective judgements involved are laid bare to scrutiny.

The work of merging reports, searching for companion reports, contacting authors, and re-analysing raw data has been time-consuming. We started with the most recent study publications to favour up-to-date data. Nevertheless, urine collection across the included studies spans 35 years, making temporal trends an important consideration. Repeated 24 h Uvol measurements in some open cohorts have shown significant change within one generation (13, 70).

Recent authoritative papers have emphasized the importance of using 24 h Uvol collections for estimating salt intake in populations (71, 72). Nationally representative “salt intake” studies constitute about 25% of all included studies in CURV-24. In retrospect, focussing solely on this subgroup of studies could have reduced workload while maintaining methodological quality and extending publication year coverage. An alternative approach would have been to involve national public health institutions and highly cited researchers within the relevant fields to identify eligible studies over the full time-range in CURV-24. Several global SRs of physiological outcomes within the Non-Communicable Disease Risk Factor Collaboration have chosen this strategy with great success (67, 73, 74). Sensitivity analyses may be able to answer whether these alternative approaches would change the risk of bias in the outcomes.

The PRISMA diagram involves some subjective judgement in defining unique studies. A few studies covered several countries, sometimes with separate protocols for data collection, but were jointly analysed in the primary report. Some reports were pooled secondary analyses of several distinct studies, each with a host of more specific companion reports. These have been split into separate studies, still retaining the name of the principal article identified through the syntax search.

The team achieved high reviewer agreement in the paired-review process: 7.1% of records had to be discussed during title and abstract screening, and just 3% in the full-text review. The lower information content on study design and covariates found in titles and abstracts may explain why the first stage required more consensus work.

### 4.2 Description of the included studies

Within the most recent 5 years of scientific publications, we have been able to identify more than 500 reports in which at least one of the CURV-24 outcomes has been measured. However, only half of these reports have resulted in study inclusion, despite extensive searches for companion reports, data sets, and unpublished results. A comparative analysis of study characteristics can indicate whether selective inclusion may have introduced bias. If so, well-conducted studies from active or well-resourced research groups may have been more likely to yield a response to our data requests. In CURV-24, the number of included primary reports published during 2020–2024 declined over time. In contrast, records retrieved during title and abstract screening showed an increasing trend across the same period. We note that collection of 24 h Uvol is often planned to answer several research questions, and measurements are regularly used in secondary analyses. Older studies may have had a longer time window for publishing results, and therefore a higher probability of at least one article reporting the desired outcome(s). Reflecting this pattern, 23% of the urine collections included in CURV-24 were performed before 2010. Temporal changes in dietary habits, hydration patterns, and body composition across populations will therefore need to be considered when interpreting the findings of the meta-analyses. Open-cohort studies with repeated waves of data collection may be particularly informative in this respect. Laboratory methods for creatinine measurement have also varied in precision over time, with international standardisation occurring mainly during the past 15 years. Both secular changes in population characteristics and changes in laboratory methodology should therefore be considered as potential contributors to heterogeneity.

Almost half of the studies had fewer than 200 participants. This may indicate that collecting 24 h Uvol is resource-demanding and seldom feasible in larger epidemiological studies. When it is done, only a fraction of the research participants is typically involved (75, 76). We are slightly surprised by the low number of hydration studies in CURV-24. It may imply that many human hydration studies are not measuring 24 h Uvol.

### 4.3 Strengths, limitations, and future directions

Data extraction and data cleaning were finalized in May 2026, and the first reports on the primary outcome are planned to be published in the coming year. Strengths of CURV-24 include broad database coverage, the comprehensive search syntax with pilot-testing of sensitivity and specificity, no language restrictions, and extensive supplementary information about each study. Of the included studies, 77% were performed after 2010. With more than 200 national populations and approximately 270,000 subjects included, it is the most comprehensive collection of its kind to date. CURV-24 capitalizes on sub-aggregated data from more than 50 unpublished raw-data sets.

The few studies from low-income and African countries limit generalizability to these parts of the world. Limited presence of certain predictor variables, missing data, uncertainty introduced by unstandardized measurements, and inconsistent reporting may hamper the quality and statistical possibilities in CURV-24. We did not run a simulation for the precision of 24 h UCrEx. In general, the between-population variance of UCrEx is smaller than for Uvol and may challenge the ability to draw firm conclusions from meta-analyses with creatinine as the outcome (42).

CURV-24 is positioned to fill a critical research gap in hydration adjustment of urinary biomarkers. The review’s prediction intervals for 24 h Uvol can quantify the uncertainty around excretion estimates derived from biomarker concentrations. It will also be possible to reassess conclusions from previous epidemiological studies that employed fixed assumptions about average 24-h urine volume or creatinine excretion, for example in iodine intake estimation (77–79). For surveys in which 24 h urine collection is not feasible, identifying simple population-level predictors may improve the accuracy of exposure and intake estimates. CURV-24 will also shed light on the limitations of applying normative physiological estimates—largely derived from high-income countries—to diverse global populations. Through a detailed assessment of risk of bias in urine collection and creatinine analysis, we aim to develop guidance on best practice, including recommendations on what and how to report. Looking ahead, linking CURV-24 outcomes to population-specific climatic data can generate hypotheses on how climate change and environmental conditions affect human hydration. Finally, CURV-24 plans to publish the variable dictionary of the full dataset and welcomes future collaboration on hydration-related meta-research (80–83).

## Data Availability

The aggregated data supporting the conclusions of this article will be made available by the authors, without undue reservation.
