# Supplementary material for: Design, methods and populations of the CURV-24 study: a global systematic review and meta-analysis of 24-h urine volume and creatinine excretion in community-dwelling adults
Source: Front Public Health. 2026 Sep 25;14:1892695. doi: 10.3389/fpubh.2026.1892695 (PMC13649961; doi:10.3389/fpubh.2026.1892695)
Supplement: Supplementary file 1 [file Data_Sheet_1.pdf]

Supplementary Material

Design, methods and populations of the CURV-24 study: A global systematic review and meta-analysis of 24-hour urine volume and creatinine excretion in community-dwelling adults

Contents

Supplementary Material 1

I) Conditions, characteristics and contexts (in)compatible with inclusion 2

II) Terms related to inclusion or exclusion of title and abstract 5

III) The final syntax for all eligible databases 6

IV) A reproduction of the data-extraction form 10

Letter to research groups asking for contextual information: 23

Introduction to the web-based questionnaire for research groups: 24

V) Items for bias assessment of outcomes in individual studies 25

I) Conditions, characteristics and contexts (in)compatible with inclusion

Update III 12.09.2024

The table below describes medical conditions, characteristics and study contexts of eligible study populations expected or encountered during the screening process and outlines the decision regarding the fulfilment of the inclusion criteria:

"A general healthy population during physiologically non-manipulated, community-dwelling, and habitual daily living"

"Yes" means the condition, characteristics or context permitted inclusion of the related population. The list is not exhaustive but provides guidance for similar conditions not listed.

**Condition, characteristic or context**	**Include as a study in the SR?**	
Age and time matched controls	Yes	
Coronary heart disease	Yes	Without symptoms or sequelae
Hypercholesterolemia	Yes	If treated and stabilized
Hyperlipidemia	Yes	If treated and stabilized
Hypertension	Yes	If well controlled and above 25 years
Hypothyroidism	Yes	If well treated without symptoms
Kidney donors	Yes	
Metabolic syndrome	Yes	If treated and stabilized
Migrants	Yes	
Nuns/Monks/religious groups	Yes	
Offspring of hypertension	Yes	
Overweight	Yes	If mean BMI < 30
Prehypertension	Yes	
Selected age range	Yes	
Specific educational groups	Yes	
Vegetarians/vegans/pescatarians	Yes	
Athletes	Yes/No	Case by case decision
During COVID-19 lockdown	Yes/No	Case by case decision
Migraine	Yes/No	Case by case decision
Nursing home residents	Yes/No	Case by case decision
Osteopenia/Osteoporosis postmenopausal	Yes/No	Case by case decision
PTSD	Yes/No	Case by case decision
Acromegaly	No	
Acute cervical spinal cord injury	No	
Alcoholism	No	
Anorexia nervosa	No	
Anosmia	No	
Apnea	No	
Asthma	No	
Atrial fibrillation	No	

Bartter syndrome	No	
Beta-thalassemia minor	No	
Cancer patients	No	
Chronic renal failure	No	
Chronic kidney disease CKD	No	
Chronic fatigue syndrome	No	
Cirrhosis	No	
Coeliac disease	No	
Congenital blindness	No	
Coronary heart disease	No	If symptoms or sequelae is persistent
Diabetes mellitus	No	Regardless of type
Diabetic kidney disease	No	
Dialysis patients	No	
Distal renal tubular acidosis	No	
Drug abuse	No	
Fractures	No	
Gitelman syndrome	No	
Gout	No	
Glomerulonephritis	No	
Heart failure	No	
Heart transplant	No	
Haemodialysis	No	
Haemorrhagic fever with renal syndrome	No	
HIV	No	
Horseshoe kidneys	No	
Hypercalciuria	No	
Hyperkalemia	No	
Hyperparathyroidism	No	
Hypertension grad II-IV	No	
Hypertension below 25 years	No	
Hypertension, gestational	No	
Hyperthyroidism	No	
Hyperuricemia	No	
Hyponatremia	No	
Hypothyroidism	No	If untreated or poorly regulated
Idiopathic hypercalciuria	No	
Idiopathic primary aldosteronism	No	
Incontinence	No	
Interstitial cystitis	No	
Isolated hematuria	No	
Kidney stones/nephrolithiasis	No	
Medullary sponge kidney	No	
Meningococcal infection	No	
Nephropathy	No	
Nephrotic syndrome	No	
Obstructive sleep apnoea	No	
Oedema	No	
Organ transplants	No	
Orthostatic hypotension	No	
Peritoneal dialysis	No	
Polycystic kidney disease	No	
Polydipsia	No	
Postural tachycardia syndrome	No	

Preeclampsia	No	
Primary aldosteronism	No	
Severe mental diseases	No	
Recurrent syncope	No	
Renal tubular acidosis	No	
Rheumatoid arthritis	No	
Short bowel syndrome	No	
Sjogren's syndrome	No	
Spaceflight	No	
Stroke	No	
Tachycardia syndrome	No	
Thalassemia	No	
Quadriplegia, paraplegia	No	

The listing is built on eligible study populations identified by Tan et al. for the project encompassing the article: M. Tan, F. J. He, C. Wang and G. A. MacGregor. Twenty-Four-Hour Urinary Sodium and Potassium Excretion in China: A Systematic Review and Meta-Analysis. J Am Heart Assoc 2019 Vol. 8 Issue 14 Pages e012923.

II) Terms for signalling inclusion and exclusion of title and abstracts

The key words, presented below, would either increase or reduce the possibility of moving a reference into full-text screening. In the Covidence environment, these words were highlighted in green and red, respectively.

Inclusion indicators

- 24h
- 24-h
- 24-hour
- twentyfour
- daily
- hour
- urine
- void
- creatinine
- excretion
- sample
- collection
- adult
- general
- healthy
- cohort
- community
- volunteers
- freeliving
- free-living
- volume
- UIE
- frequency-volume-chart
- controls
- hours
- 24
- method
- methods
- Cortisol
-

Exclusion indicators

- child
- children
- pediatric
- paediatric
- neonate
- school
- schools
- patient
- patients
- hospital
- intensive care unit
- ICU
- tertiary care
- syndrome
- fever
- edema
- oedema
- vomit
- nausea
- anorexia
- diarrhoea
- diarrhea
- dialysis
- haemodialysis
- hemodialysis
- intravenous
- infusion
- injection
- chronic kidney disease
- CKD
- polycystic kidney disease
- PKD
- polyuria
- heart failure
- diabetes
- urolithiasis
- kidney stone
- incontinence
- Tolvaptan
- lithium
- Desmopressin
- Terlipressin
- Furosemide
- diuretics
- nephrolithiasis
- nephritis
- nephropathy
- glomerulopathy
- Wilson
- SLE
- overactive bladder
- thyroidism
- hypothyroidism
- hyperthyroidism
- hypoparathyroidism
- hyperparathyroidism
- IgAN
- stroke
- Spot

III) The final syntax for all eligible databases

The following databases were searched for records between 1990 and 2024:

Database	Number of retrieved records
MEDLINE (Ovid)	12604
Embase (Ovid)	15712
Global health (Ovid)	3722
Cochrane Library (ref fra ICTRP, CT.gov er ikke tatt med	3729
Web of Science - All indexes	13039
Global Index Medicus – World Health Organization	1577
Number of records before deduplication:	50085
Number of records after deduplication:	21891
Number of records including supplementary search	22676
**Number of records after 01.01.2020**	**5217**

All searches were performed on 02.01.2024, with supplementary search on 12 Sep 2024 which added 785 new records after deduplication.

Strategies where developed by Hilde Iren Flaatten, Sara Clarke, and Skjalg Kalvik, University of Oslo Library of Medicine and Science.

Database(s): **Ovid MEDLINE(R) ALL**

Search Strategy:

#	Searches	Results
1	((24hour* or twentyfourhour* or 24h* or 24 h* or twenty four hour* or twentyfour hour* or day or daily) adj3 (urin* or void* or volume*)).tw,kw,kf.	32531
2	(("24" or twentyfour or twenty four) adj2 (hour* or h) adj3 (urin* or void* or volume*)).tw,kw,kf.	21211
3	((("24" or twentyfour or twenty four) adj2 (hour* or h) adj2 (creatinin* or sodium* or na or NaCl or natrium* or salt* or potassium* or k or kalium or iodine*)) and (collect* or excret*)).tw,kw,kf.	2256
4	(((24hour* or twentyfourhour* or 24h* or 24 h* or twenty four hour* or twentyfour hour* or day or daily) adj2 (creatinin* or sodium* or na or NaCl or natrium* or salt* or potassium* or k or kalium or iodine*)) and (collect* or excret*)).tw,kw,kf.	3901
5	(urin* adj2 (cortisol* or iodine excretion* or sodium excretion*)).tw,kw,kf.	8442
6	(frequency volume chart* or urin* free cortisol* or urin* iodine excretion* or ((24hour* or twentyfourhour* or 24h* or "24 h*" or "twenty four hour*" or twentyfour hour* or day or daily or urin*) and (uie or ufc))).tw,kw,kf.	3242
7	or/1-6	40416

8	Pregnancy/ or Breast Feeding/ or Pregnant Women/ or (pregnan* or breastfeed* or breastfed or breast feed* or breast fed).tw,kw,kf.	1190041
9	7 and 8	2255
10	Healthy Volunteers/ or (population* or volunteer* or epidemiolog* or healthy* or normal* or participant* or general or communit* or municipality or municipalities or city or cities or town* or suburb* or township*).tw,kw,kf.	7505392
11	7 and 10	19005
12	(neonates/ or premature infants/ or infant, newborn/ or infant/ or child, preschool/ or pediatrics/ or child/ or puberty/ or adolescent/ or schools, nursery/ or child day care centers/ or child care/ or (newborn* or new born* or baby or babies or infan* or neonat* or child* or minors or under age or kid or kids or youngster* or toddler* or preschool* or pre school* or kindergarten* or schoolchild* or school age or girl* or boy* or pediat* or paediat* or preadolesc* or adolescen* or juvenile or prepubescen* or prepuberty* or puberty or pubescen* or teen*).ti,kf.) not (adult/ or exp aged/ or middle aged/ or young adult/ or men/ or women/ or pregnant women/ or (adult* or men or women or man or woman or participant* or middle aged or geriatric* or senior* or senium or elderly or ((old* or elder*) adj (age or people or subject* or population* or person*)) or very old or septuagenarian* or octagenarian* or octogenarian* or nonagenarian* or centarian* or centenarian*).ti,kf.)	2495888
13	9 or (11 not 12)	18399
14	limit 13 to yr="1990 -Current"	15074
15	(exp Animals/ or exp Animal Experimentation/ or exp models, animal/ or exp Animal Diseases/ or exp Animals, Genetically Modified/ or exp Veterinary Medicine/ or Hospitals, Animal/ or (rat or rats or animal or animals or veterinary or mice or mouse or rabbit or rabbits or murine or dog or dogs or invertebrate or invertebrates or vertebrate or vertebrates or cat or cats or rodent or rodents or mammal or mammals or gorilla or gorillas or monkey or monkeys or canine or canines or bird or birds or hamster or hamsters or primate or primates or horse or horses or avian or avians or equine or equines or macaque or macaques or diptera* or amphibian or amphibians or snake or snakes or feline or felines or macaca or macacas or proteus or pigeon or pigeons or bat or bats or chimpanzee or chimpanzees or baboon or baboons or canis or reptile or reptiles).ti,kf.) not (Humans/ or exp Human Experimentation/ or men/ or women/ or pregnant women/ or (human or humans or patient or patients or person or persons or man or men or woman or women).ti,kf.)	5351280
16	14 not 15	13455
17	limit 16 to (address or autobiography or bibliography or biography or case reports or comment or consensus development conference or consensus development conference, nih or dictionary or directory or editorial or lecture or legal case or letter or news or newspaper article or video-audio media or webcast or festschrift or historical article or interactive tutorial or patient education handout or personal narrative)	851
18	16 not 17	12604

Database(s): **Embase Classic+Embase**

Search Strategy:

#	Searches	Results
1	urine volume/	30367
2	(24hour* or twentyfourhour* or 24h* or 24 h* or twenty four hour* or twentyfour hour* or day or daily).tw,kf.	3393766
3	(("24" or twentyfour or twenty four) adj2 (hour* or h)).tw,kf.	676718
4	1 and (2 or 3)	10912
5	((24hour* or twentyfourhour* or 24h* or 24 h* or twenty four hour* or twentyfour hour* or day or daily) adj3 (urin* or void* or volume*)).tw,kw,kf.	56143
6	(("24" or twentyfour or twenty four) adj2 (hour* or h) adj3 (urin* or void* or volume*)).tw,kw,kf.	33303
7	((("24" or twentyfour or twenty four) adj2 (hour* or h) adj2 (creatinin* or sodium* or na or NaCl or natrium* or salt* or potassium* or k or kalium or iodine*)) and (collect* or excret*)).tw,kw,kf.	3299
8	(((24hour* or twentyfourhour* or 24h* or 24 h* or twenty four hour* or twentyfour hour* or day or daily) adj2 (creatinin* or sodium* or na or NaCl or natrium* or salt* or potassium* or k or kalium or iodine*)) and (collect* or excret*)).tw,kw,kf.	6215
9	(urin* adj2 (cortisol* or iodine excretion* or sodium excretion*)).tw,kw,kf.	11979
10	(frequency volume chart* or urin* free cortisol* or urin* iodine excretion* or ((24hour* or twentyfourhour* or 24h* or 24 h* or twenty four hour* or twentyfour hour* or day or daily or urin*) and (uie or ufc))).tw,kw,kf.	5024
11	or/5-10	67203
12	4 or 11	74093
13	exp pregnancy/ or breast feeding/ or pregnant woman/ or (pregnan* or breastfeed* or breastfed or breast feed* or breast fed).tw,kw,kf.	1307715
14	12 and 13	3877
15	normal human/ or (population* or volunteer* or epidemiolog* or healthy* or normal* or participant* or general or communit* or municipality or municipalities or city or cities or town* or suburb* or township*).tw,kw,kf.	10659609
16	12 and 15	36544
17	(exp child/ or adolescent/ or pediatrics/ or nursery school/ or child care/ or infant care/ or kindergarten/ or (newborn* or new born* or baby or babies or infan* or neonat* or child* or minors or under age or kid or kids or youngster* or toddler* or preschool* or pre school* or kindergarten* or schoolchild* or school age or girl* or boy* or pediat* or paediat* or preadolesc* or adolescen* or juvenile or prepubescen* or prepuberty* or puberty or pubescen* or teen*).ti,kf.) not (adult/ or exp aged/ or middle aged/ or young	3196996

	adult/ or pregnant woman/ or (adult* or men or women or man or woman or participant* or middle aged or geriatric* or senior* or senium or elderly or ((old* or elder*) adj (age or people or subject* or population* or person*)) or very old or septuagenarian* or octagenarian* or octogenarian* or nonagenarian* or centarian* or centenarian*).ti,kf.)	
18	14 or (16 not 17)	34941
19	limit 18 to yr="1990 -Current"	28549
20	(exp animal/ or exp animal experiment/ or exp animal model/ or nonhuman/ or exp animal disease/ or exp veterinary medicine/ or veterinary clinic/ or (rat or rats or animal or animals or veterinary or mice or mouse or rabbit or rabbits or murine or dog or dogs or invertebrate or invertebrates or vertebrate or vertebrates or cat or cats or rodent or rodents or mammal or mammals or gorilla or gorillas or monkey or monkeys or canine or canines or bird or birds or hamster or hamsters or primate or primates or horse or horses or avian or avians or equine or equines or macaque or macaques or diptera* or amphibian or amphibians or snake or snakes or feline or felines or macaca or macacas or proteus or pigeon or pigeons or bat or bats or chimpanzee or chimpanzees or baboon or baboons or canis or reptile or reptiles).ti,kf.) not (exp human/ or exp human experiment/ or pregnant woman/ or (human or humans or patient or patients or person or persons or man or woman or men or women).ti,kf.)	8141975
21	19 not 20	25419
22	limit 21 to (books or chapter or conference abstract or editorial or letter or note or "preprint (unpublished, non-peer reviewed)" or tombstone)	9707
23	21 not 22	15712

IV) A reproduction of the data-extraction form in Covidence

DATA EXTRACTION

General information

Main field of research

- ○ Salt intake
- ○ Kidney-Uro-Genital
- ○ Hydration
- ○ Iodine nutrition
- ○ All other micronutrients
- ○ Hormones/neurotransmitters
- ○ Heavy metals
- ○ Environmental substances
- ○ Other

Clear above selection

First author name

First name, family name

E-mail

UR

Key researcher name

First name, family name

E-mail

UR

Characteristics of included studies

Aim of the study

Is 24h Uvol or 24h creatinine excretion a defined outcome in the study?

- ○ Yes
- ○ No
- ○ Unclear

Clear above selection

Study design

- ○ Cross sectional study
- ○ Longitudinal prospective/Cohort study
- ○ Case-control study
- ○ Clinical trial
- ○ Non-randomised experimental study
- ○ Diagnostic test accuracy study
- ○ Other

Participant selection

Recruitment strategy

Sampling frame (population) description

UR

Sampling frame population

Always free-living (if not:exclude)

- ○ General
- ○ General, healthy selection
- ○ Selected with a mild disease level
- ○ Unclear

Clear above selection

Participant inclusion criteria

Participant exclusion criteria

Smaller exceptions from the inclution criterias or NA/UR

Sampling method

- ○ Random
- ○ Quasi-random
- ○ Systematic
- ○ Convenience
- ○ Unclear

Clear above selection

Participant selection bias

UR

	Total participant number
Total Invited	
Total after Declined	
Total after Excluded	
Total after Drop outs	
Total with outcome data	

Balanced subgroup coverage

- ○ Yes
- ○ No
- ○ Unclear

Clear above selection

Overall participant selection bias (internal + external validity): Does the outcome data subgroup represent the general population of interest in the SR?

- ○ Yes (Acceptable)
- ○ No
- ○ Unclear

Place and time

Country in which the study was conducted

UR

Region (sub-national) of urine sampling

UR

Place/city of urine sampling

UR

Year of the first urine sampling

UR

Start month (01-12)

UR

Year of last urine sampling

UR

End month (01-12)

UR

Sample population characteristics

Full population data

Are there outcome data for the full sample (population)?

If Yes, populate the characteristics, if No - go to subpopulation section

- ○ Yes
- ○ No

Clear above selection

Sex

- ○ M
- ○ F
- ○ Both
- ○ Unclear

Clear above selection

Fertility status for females

- ☐ Pregnant
- ☐ Breastfeeding
- ☐ Nonpregnant and nonbreastfeeding
- ☐ Unclear

Clear above selection

SES-compared with the country's/locations general population

- ☐ High
- ☐ Medium
- ☐ Low
- ☐ Unclear

Clear above selection

Ethnicity, specify

UR

Outcome

Outcome variables of interest

If 24h Uvol is reported, U-creatinine concentration is valuable as well (/L), otherwise U-creatinine needs to be excretion/24h.

	Measurement unit (if other)	n	n missing	P0	P25	Median	Mean	P75	P100	SD	SEM	95% CI low	95% CI high
24h Uvol (L)													
Urinary creatinine (mmol/24h)													
24h Urinary osmolality - (mOsmol/kg)													
24h Urinary spesific gravity (1.001-1.040)													
24h Urine colour (8 scale)													
U-Na excretion (mmol/24h)													
U-K excretion (mmol/24h)													
Urinary iodine excretion (microg/24h)													

Age and anthropometry

	Measurement unit (if other)	n	P0	P25	Median	Mean	P75	P100	SD	SEM
Age (y)										
Height (m)										
weight (kg)										
BMI (kg/m2)										

Other defined co-variables

free text or NA

Subpopulations

Number of subpopulations with primary/secondary outcome

0 - skip and go to Quality Control. > 0 - in succession of diminishing n

Subpopulation 1

Place - as detailed as possible

Sex

- ○ M
- ○ F
- ○ Both
- ○ Unclear

Clear above selection

Fertility status for females

- ☐ Pregnant
- ☐ Breastfeeding
- ☐ Nonpregnant and nonbreastfeeding
- ☐ Unclear

Clear above selection

SES - compared with the country's/citys general population

- ☐ High
- ☐ Medium
- ☐ Low
- ☐ Unclear

Clear above selection

Ethnicity, specify

UR

The extraction form for subpopulations included the same matrix for age, anthropometrics, outcomes and covariates as shown for the "total population". It was possible to extract data for up to 24 subpopulations per study and form.

Quality control of Uvol collection

Collecting days of the week

- ○ 5/7
- ○ 7/7
- ○ Unclear

Clear above selection

Collection permitting habitual living?

- ○ Yes
- ○ No
- ○ Unclear

Clear above selection

Correct 24h Uvol collection method and measurement?

- ○ Yes
- ○ No
- ○ Unclear

Clear above selection

Instruction in urine collection

- ☐ Written/oral/video/demonstration
- ☐ Unclear
- ☐ None

Clear above selection

Testing of participant comprehension and/or collection ability

- ○ Yes
- ○ No
- ○ Unclear

Clear above selection

Participant support during 24h collection available?

- ○ Yes
- ○ No
- ○ Unclear

Clear above selection

Observed urine collection timing?

- ☐ Start
- ☐ End
- ☐ None
- ☐ Unclear

Clear above selection

Uvol values excluded/adjusted?

- ○ Yes
- ○ No
- ○ Unclear

Clear above selection

Uvol values excluded/ values adjusted

	Exclusion rule	Adjustment rule
Volume		
creatinine amount		
SG/Osmolality/Urine colour		
PABA recovery or similar		
Duration		
missed voids		

Other - describe

Sensitivity analysis/reliability assessment for population volume?

- ○ Yes
- ○ No
- ○ Unclear

Clear above selection

Is the "true" mean 24h Uvol from collection among the participants likely towards

consider the collection protocol and bias adjustments

- ○ Same value
- ○ Lower value
- ○ Higher value
- ○ Don't know

Clear above selection

Summary risk - 24h Uvol collection bias

- ○ Low
- ○ Unclear
- ○ High

Quality control of creatinine analysis

Was creatinine values reported?

- ○ Yes
- ○ No

Clear above selection

Sound handling/laboratory manual followed

- ○ Yes
- ○ No
- ○ Unclear

Clear above selection

Laboratory analysis line/equipment described?

- ○ Yes
- ○ No
- ○ Unclear

Clear above selection

Method of creatinine analysis

- ○ Jaffe
- ○ Jaffe compensated
- ○ Enzymatic dry
- ○ Enzymatic liquid
- ○ Mass Spectrometry
- ○ Other

Clear above selection

Other Creatinine analysis method - describe

Instrument manufacturer

UR

Instrument Analyzer name

UR

Laboratory uncertainty reported?

- ○ Yes, error
- ○ Yes, bias
- ○ Yes, error and bias
- ○ No

Clear above selection

Write error values

UR

Write bias values

UR

Acceptable laboratory uncertainty?

Study CV of +/- 5%. General mean bias range of +/- 6%

- ○ Yes
- ○ No
- ○ Unclear

Clear above selection

Sensitivity analysis/reliability assessment for population creatinine concentration/excretion?

- ○ Yes
- ○ No
- ○ Unclear

Clear above selection

The "true" mean creatinine concentration in the participants likely towards

- ○ Same value
- ○ Lower value
- ○ Higher value
- ○ Don't Know

Summary risk for creatinine concentration measurement bias

- ○ Low
- ◉ Unclear
- ○ High

Potential for comparing gold standard and spot urine

24 hour Uvol x 2 within 6 months collected in the study?

○ Yes
○ Not likely
○ Unreported

Clear above selection

Spot urine sample from same period collected?

○ Yes
○ Unreported

Clear above selection

Urine biobanking likely?

○ Yes
○ No
○ Unreported

Clear above selection

Analyzed for Iodine concentration or excretion?

○ Yes
○ Unreported

Clear above selection

Miscellaneous

Citations (DOI) from this study to explore further

Use new line to delimit if >1. Use the comment field for explanations

Any special reasons to contact the authors?

General notes from the reviewer about this study

Letter to research groups asking for contextual information:

Dear colleague,

As you may know from earlier correspondence, I am currently leading a study aimed at collecting mean/median 24-hour urine volume and mean/median 24-hour creatinine excretion from primary studies (CURV-24). The purpose is to determine whether urine biomarkers can be meaningfully compared across diverse populations when measured as a plain concentration or concentration adjusted per gram of creatinine. We believe this endeavor may be important for many research fields using biomarkers in urine. You can find information about the systematic review here: OSF | 24-hour urine volume and 24-hour urine creatinine excretion in general adult populations, a systematic review and meta-analysis. and the protocol itself: OSF | uvol24h_doc_protocol_20240202.pdf

You have been involved in at least one study included in CURV-24. In conjunction with extracting the CURV-24 outcomes and co-variables, which may not be the same as your study's outcome(-s), we are undertaking an assessment of the risk of bias. We are contacting you to request additional contextual information beyond what could be obtained from the published article(s) related to your study. With the additional information, we may be able to elevate your study to "high trust in the reported data". We believe this is important for you when we cite your study, and for us – important for how normative our conclusions can be. Bear with us if you have shared some of this information before.

Here is the web-based questionnaire https://nettskjema.no/a/conxinfo. The information and your personal data will be stored safely and not be shared with third parties. The questionnaire itself should not take too much of your time, given that you have the information at hand. Attached is a pdf document listing your name (first name in alphabetic order). To the right, you will find the title of the primary article representing your included study, the first author/publication year and a CURV-24 generated ID number. This ID number will identify the questionnaire answers and shall be filled in at the start. If you are listed as contact person for more than one study, please send us one form for each. If you believe another person is better suited to fill in the form, please forward this email to that person and keep me in the cc field 😊

We will acknowledge everyone who supports the study with data, so please add your current affiliations.

We hope to hear from you soon, ideally within 10 days, as we are about to close the data extraction phase. If you have any questions, or struggle with the questionnaire, please do not hesitate to reach out to me through this e-mail.

Cordially,

(The web-based questionnaire contained the same items as the data-extraction form when clarifying the context of the study and the details related to the bias assessment.)

Introduction to the web-based questionnaire for research groups:

Dear colleague,

Thank you for your willingness to provide further details about your study! The questionnaire below was designed to clarify the methods used for selecting, collecting and measuring participants' 24-hour urine volume and, where applicable, the analysis of urinary creatinine.

This data will only be used to do a quality appraisal in CURV-24, and not shared with third parties. Your form entry will be deleted when the information is transferred to the main dataset for analysis. Your name and email address will not be part of the data set.

With the additional contextual information, we may be able to elevate your study to a "high trust in the reported data" pertaining to the outcomes CURV-24 are investigating.

Under each item, please write directly into the textbox or mark the option that best describes the alternatives. (Item numbers correspond to our comprehensive data collection form - this constituting a subset.)

We understand that it might not be possible to answer every question - any new information is important and valuable.

Thank you again for supporting this systematic review.

V) Items for bias assessment of outcome in individual studies

	Bias assessment items for 24h Uvol and creatinine excretion	Necessary for objective low bias score?	Score weight if yes
	**24h Urine Volume collection**		
1.	Allocation to 7 days a week sampling	No	1
2.	The organisation of collection (including equipment) allowing habitual living	Yes	1
3.	Correct 24h Uvol collection method and measurement	Yes	2
4.	Instruction in urine collection (written/oral/video/demo)	Yes	1
5.	Testing of participant comprehension and their collection ability	Yes	1
6.	Participant support during urine collection available	No	1
7.	Observed collection start or end or (both)	No	1 (2)
8.	**Exclusion of urine volume observations based on**		
a.	Population specific Uvol min and/or max limits OR	Yes of the one	2
b.	Population specific 24h Creatinine excretion range OR	with the	1
c.	SG/Osmolality/Urine colour cut-offs OR	highest score	1
d.	PABA or similar biomarkers of collection OR		1
e.	Time-collection duration in hours AND missed voids ratio		2
		AND/OR	
9.	**Adjustment of reported urine volume based on**		
a.	Population specific Uvol min and/or max limits OR	Yes of the one	2
b.	Population specific 24h Creatinine excretion range OR	with the	1
c.	SG/Osmolality/Urine colour cut-offs OR	highest score	1
d.	PABA or similar biomarkers of collection OR		1
e.	Time-collection duration in hours AND missed voids ratio		2
10.	Sensitivity analysis or reliability assessment for volume distribution in the primary study	No	1
11.	**Creatinine (building on 1-10)**		
a.	Sound handling/laboratory manual followed	Yes	2
b.	Laboratory analysis line/equipment (ISO) certified	Yes	2
c.	Creatinine - laboratory quality control of bias and error measures acceptable	Yes	2
12.	Sensitivity analysis or reliability assessment for creatinine distribution in the primary study	No	1

The information was recorded in the data-extraction form. This scorecard is being used as a guide to the weighing of information, and what the team consider necessary to give a "low risk of bias" score for the three domains of bias assessment.
